# Human Evaluation of the Glu298Asp Polymorphism in NOS3 Gene and its Relationship with Onset age of ESRD in Iranian Patients Suffering from ADPKD

**Published:** 2012

**Authors:** Negin dasar, Sayyed Mohammad Hossein Ghaderian, Eznollah Azargashb

**Affiliations:** 1 *Pardis International Unit, University of Guilan , Rasht, Iran.*; 2 *Department of Medical Genetics, Faculty of Medicine, Shahid Beheshti University of Medical Sciences and Health Services, Tehran, Iran.*; 3 *Department of Health & Community Medical, Faculty of Medicine, Shahid Beheshti University of Medical Sciences and Health Services, Tehran, Iran.*

**Keywords:** ADPKD, ESRD, NOS3, Glu298Asp polymorphism

## Abstract

One of the most striking features in autosomal dominant polycystic kidney disease (ADPKD) is the difference at onset age of end-stage renal disease (ESRD). Modifier genes may play a role in this phenotypic variability. The mutated nitric oxide synthase 3 gene (NOS3), have a modifier effect on the severity of ADPKD by impairment of NOS3 activity and decreasing of renal vascular nitric oxide production and, subsequently, reduced kidney function. In order to test this hypothesis, we investigated the relationship between Glu298Asp polymorphism in exon 7 of this gene and ESRD in ADPKD patients refered from Shahid Labbafi Nedjad Hospital in Tehran. The polymorphism was examined by PCR, followed by RFLP (with MboI) in three groups of ADPKD with ESRD; ADPKD without ESRD patients and normal individual as the cases, case-controls and controls, respectively. The frequencies of GG, GT, and TT genotypes in cases were 66.7%, 33.3% and 0%, in case-controls were 78.6%, 19%, 2.4%, and in controls were 64.3%, 35.7% and 0%, respectively. Our findings revealed that there was no significant difference in the genotype frequency of NOS3 gene in ADPKD patients (p=0.311).The age of onset of ESRD in ADPKD patients, harbouring the T allele of this polymorphism, was two years lower than G/G patients, but this difference was not significant (*p* =0.641). In conclusion, our results suggest that there is no evidence of relationship between Glu298Asp polymorphism and onset age of ESRD in Iranian ADPKD patients.

Autosomal dominant polycystic kidney disease (ADPKD) is the most prevalent, potentially lethal, inherited kidney disease that its treatment is limited to reducing symptoms and mortality (1). It is characterized by the formation of multiple cysts in the cortex and medulla of both kidneys. ADPKD patients proceed to renal failure because of multiple renal cyst development and a decrease in functional nephrons due to renal parenchymal atrophy and fibrosis (2). Renal stones, urinary tract infections, hematuria and proteinuria are the most important renal manifestations of ADPKD (3). Other manifestations include cysts development in the liver, pancreas and ovaries, cerebral aneurysms and cardiac valve abnormalities (4). Hypertension is the most frequent complication among ADPKD patients, occurring in approximately 60% of the patients (5).

By the age of 60 years, about half of the ADPKD patients have progressed to end-stage renal disease (ESRD) and require renal replacement therapy (dialysis or a kidney transplant) to maintain life (6). One of the most striking features in ADPKD is the substantial variability in the severity of renal phenotype, primarily assessed by the age at ESRD. In some individuals, kidney cysts are present in early childhood and progression to ESRD occurs before 40 years of age, whereas in others, renal function remains unimpaired throughout life. This variability is observed among families, family members and even dizygotic twins (7-8). Although genetic and allelic heterogeneity explain part of this variability (9-10), significant intrafamilial differences at onset age of ESRD indicate that genetic modifiers and the environment can influence renal phenotype. Therefore, several studies have been performed to investigate other genes that may be associated with ESRD; and some cases have reported a significant correlation; such as modifier effect of nitric oxide synthase 3 (NOS3) gene in ESRD (11-12). Nos3 protein is an enzyme which catalyzes production of NO from L-arginine in endothelial cells. In this process, oxygen and NADPH are necessary as cofactor (13-14).

NO generated within vascular endothelial cells diffuses into vascular smooth muscle cells and causes vasodilation by stimulation of the guanylate cyclase pathway and the consequent generation of cyclic guanosine monophosphate (cGMP). This process is termed endothelium-dependent vasodilation (15). Generation of nitric oxide (NO) and development of end stage renal disease (ESRD) have been recently associated with the 298Asp substitution in the endothelial NO synthase (eNOS) gene (16-17). The same association was recently reported in patients with ESRD due to polycystic kidney disease (18). Therefore, the purpose of the present study was to investigate the Glu298Asp polymorphism in Iranian ESRD patients suffering from ADPKD.

## Materials and Methods

The ADPKD patients were selected from the patients referred to Shahid Labbafi Nedjad Hospital in Tehran and consisted of 42 ADPKD patients with ESRD as cases, 42 ADPKD patients without ESRD as case-controls. The Glu298Asp polymorphism was then compared with that of 42 unrelated healthy individuals as controls. ADPKD patients were recognized by ultrasonography and the diagnosis of ESRD was established on the basis of their need to renal replacement therapy (dialysis or renal transplantation).

**Fig 1:**
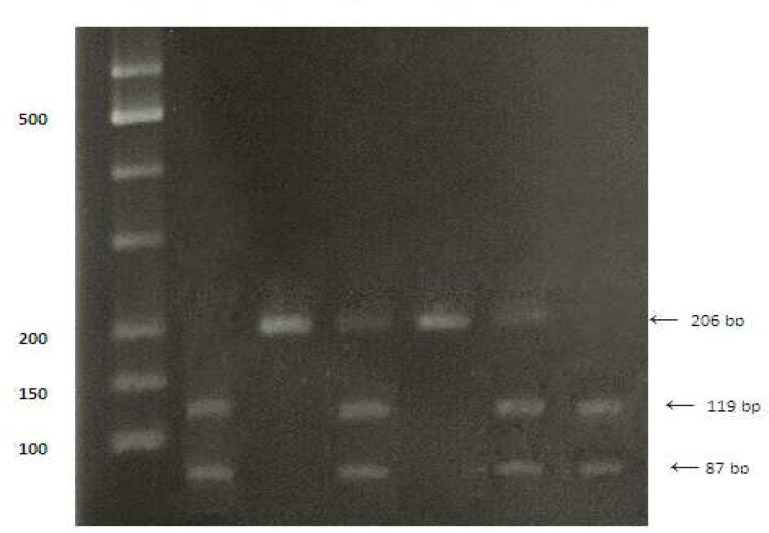
Bi-allelic polymorphism in exon 7 of the NOS 3 gene detected by MboI restriction endonuclease digestion of the 206 bp PCR product. Lane 1 show restriction patterns corresponding to homozygosity for Asp298; lane 2 show restriction patterns corresponding to homozygosity for Glu298, lane 3 show restriction patterns corresponding to heterozygosity; lane 4 is a GG homozygote ,lane 5 is a heterozygote and lane 6 is a TT homozygote.M is marker

After completing questionnaire form and taking written consent, blood samples were taken from each individual. Genomic DNA was extracted from each blood sample using Bioneer DNA extraction kit (Cat.No.K3032) according to the manufacturer’s protocol. Detecting Glu298Asp polymorphism of the NOS3 gene was performed by PCR amplification of exon 7 with the primers 5^'^-CATGAGGCTCAGCCCCAGAAC-3^'^ (sense) and 5^'^-AGTCAATCCCTTTGGTGCTCAC-3^'^ (antisense) followed by MboI restriction endonuclease digestion and resolution by electrophoresis on a 2% agarose gel. The PCR condition was 35 cycles consisting of denaturation at 95°C for 30 second, annealing at 63°C for 30 second, and extension at 72°C for 30 second. The 206 bp PCR product is cleaved into 119 bp and 87 bp fragments in the presence of a T at nucleotide 894 of the gene. All data were analysed using the SPSS statistical software (version 16) (Fig. 1).

**Fig 2:**
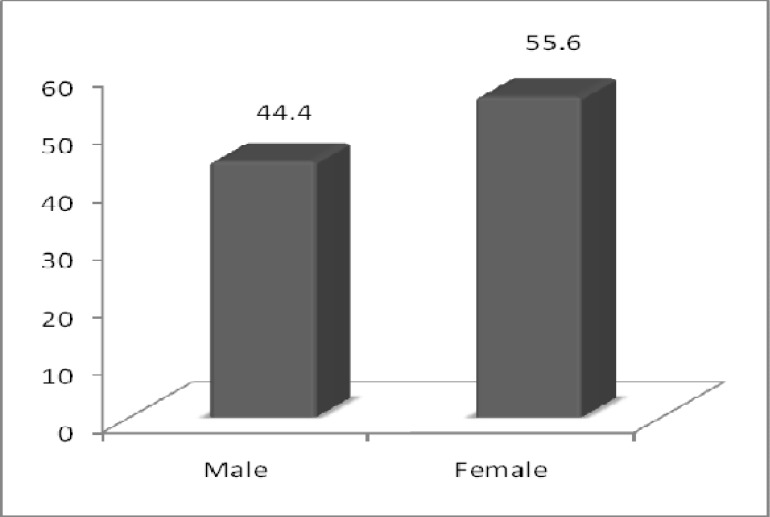
Comparing the frequency of sex in the studied groups

**Fig 3:**
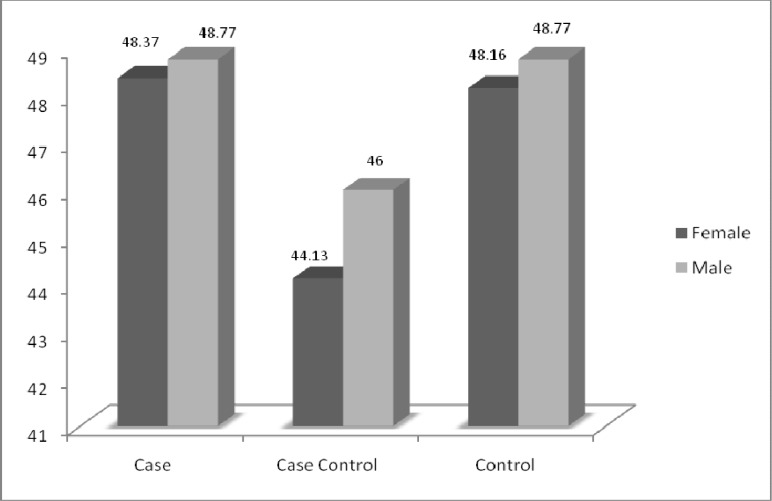
Comparing the mean age in the studied groups

**Fig 4:**
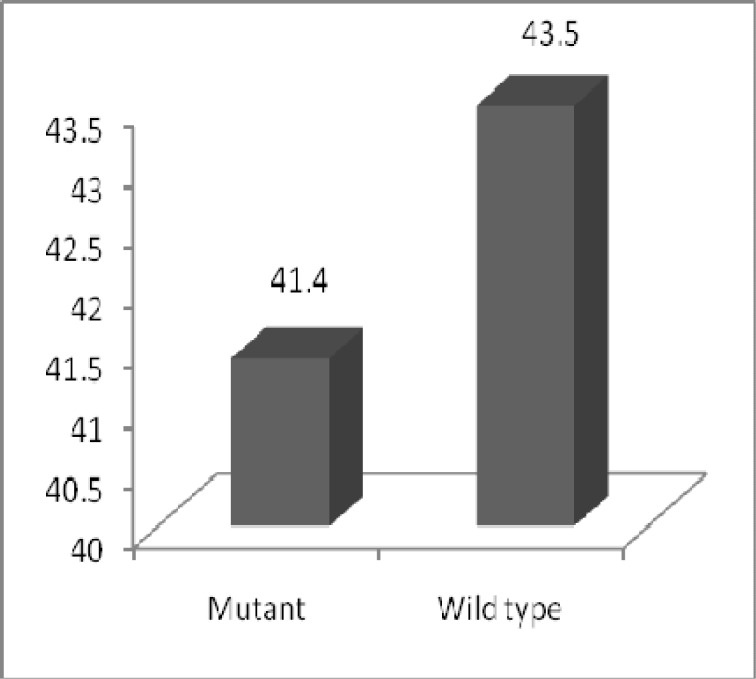
Comparing The mean age of onset of ESRD Between wild type and mutant patients for Glu298Asp polymorphism in case group.

## Results

There is no statistically significant difference between sex and medium age in studied population (Fig. 2, 3). 

The frequencies of Glu/Glu, Glu/Asp and Asp/Asp genotypes were 66.7%, 33.3% and 0% in case group, 78.6%, 19% and 2.4% in case-control group and 64.3%, 35.7% and 0% in control group. The Genotypes frequencies were not significantly different in the three groups (Table 1). Likewise, in ADPKD patients belonging to the case group that was harbouring the Asp allele of the Glu298Asp polymorphism, was 2 year lower the age of ESRD comparing to (Glu/Glu) GG Genotype patients; although there was no statistically significant difference between them (*p=*0.641) (Fig. 4).

**Correlations between Glu298Asp polymorphism and clinical parameters in ADPKD**

**Fig 5:**
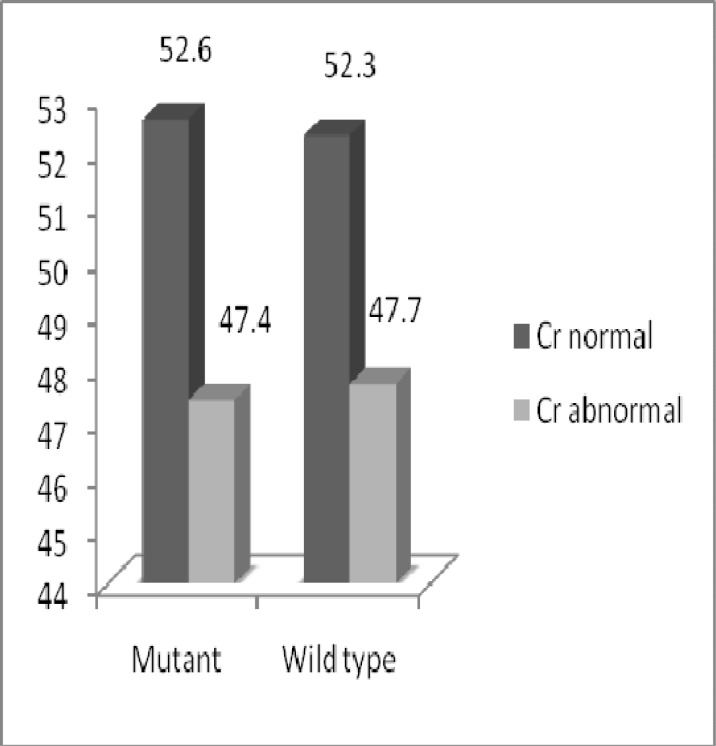
Comparison of creatinine between individuals, who were T allele carrier (mutated) and individuals, who had wild type G allele in three groups

**Fig 6:**
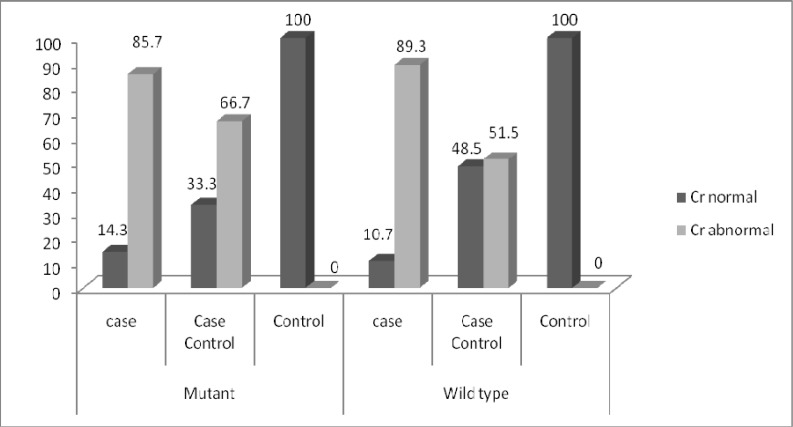
Comparison of creatinine between individuals, who were T allele carrier (mutated) and individuals, who had wild type G allele in case, case-control and control groups

Out of 126 individuals, 38 of them were carrier of T allele. Eighteen of those carriers had abnormal creatinine (Cr>1.4 mg/dl) and 20 of them had normal creatinine (Cr<1.4 mg/dl). Out of 88 individuals, who had wild type G allele, 42 had abnormal creatinine and 46 had normal creatinine. No significant differences were found in individuals due to creatinine level (*p=*0.563) (Fig. 5). In case group out of 14 individuals, carrier, of T allele 12 had abnormal creatinine. Twenty five case individuals and 17 case-control individuals, had wild type G allele and abnormal creatinine (Fig. 6).

**Table 1:** Genotype frequencies of Glu298Asp polymorphism of NOS3 gene in case, case-control and control groups

Gene	Genotype	Cases (n=42) N (%)	Case-Controls (n=42) N (%)	Controls (n=42) N (%)	P value
NOS3	G/G	(66.7) 28	33 (78.6)	27 (64.3)	0.311
	G/T + T/T	(33.3) 14	9 (21.4)	15 (35.7)	

**Fig 7:**
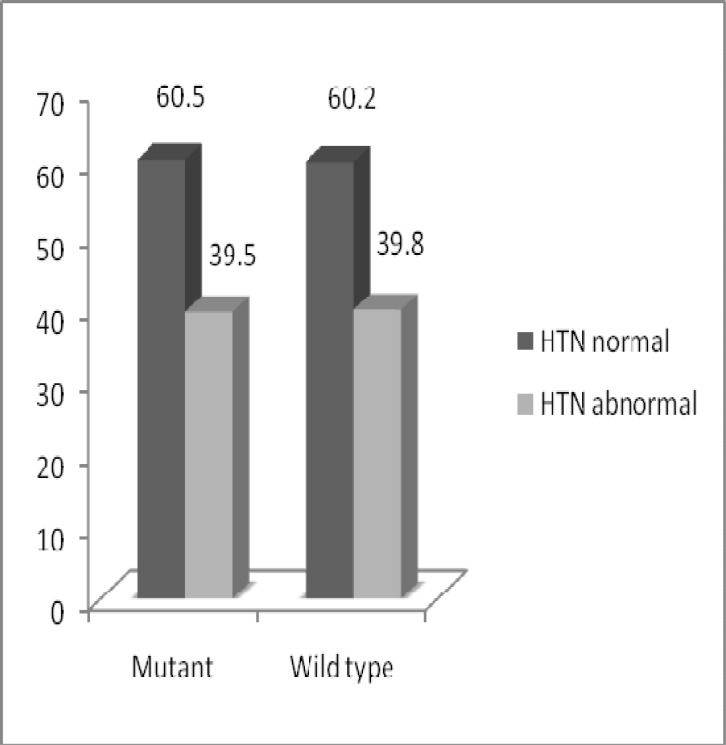
Comparison of blood pressure between individuals, who were T allele carrier (mutated) and individuals, who had wild type G allele in three groups

**Fig 8:**
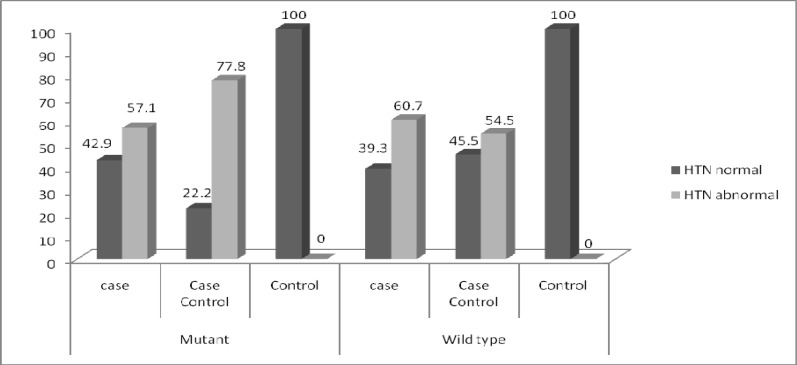
Comparison of blood pressure between individuals, who were T allele carrier (mutated) and individuals, who had wild type G allele in case, case-control and control groups

Out of 38 individuals,carrier of T allele, 15 had high blood pressure (>90/140 mm Hg) and out of 88 individuals, who had wild type G allele, 35 had high blood pressure. No significant differences were found in individuals due to blood pressure (*p=*0.568) (Fig. 7). Eight individuals from case group and nine individuals from case-control group,had T allele (mutated) and high blood pressure (Fig. 8).

In this study, relationship between Glu298Asp polymorphism and other clinical parameters related to ADPKD including hematuria, kidney stones, BUN, PLT, WBC, HDL/LDL, FBS, hemoglobin, sodium, potassium, calcium, phosphorus, triglyceride, and cholesterol were investigated. No significant differences were found in individuals due to these parameters.

## Discussion

Investigation of Glu298Asp polymorphism in NOS3 gene and its relationship with onset age of ESRD in ADPKD patients, was carried out in Shahid Labbafi Nedjad Hospital, Tehran. Fourthy two ADPKD patients who used renal replacement therapy were regarded as the case group, Fourthy two ADPKD patients who didn’t use the renal replacement therapy were regarded as the case-control group and Fourthy two individuals without ADPKD were approached as the control group. Our results showed that there was no significant relationship among the three groups due to Glu298Asp polymorphism. Regarding relationship between T allele presence and the lowering age of ESRD, in this study there were no differences between case group of ADPKD patients with T allele for Glu298Asp polymorphism and (Glu/Glu) GG genotype patients. 

In many studies Glu298Asp polymorphism of NOS3 gene has been associated with three conditions characterized by endothelial dysfunction, hypertension, *myocardial infarction,* and carotid atherosclerosis (19-21). Some studies also referred to endothelial dysfunction in Nitric oxide release and the association of Glu298Asp polymorphism with NO production and renal dysfunction in patients with ADPKD (18, 22-23). A molecular basis for the effect of the Glu298Asp polymorphism was provided by the demonstration of decreased enzymatic activity and modified expression of eNOS in renal arteries from patients harbouring the Asp allele (24). The substitution of G allele with T in exon 7 (894 G/T) results in the conversion of glutamate to aspartate at position 298 in the eNOS protein. Based on the investigations of the mechanisms that cause the polymorphism to decrease the eNOS activities, the most probable one turned out to be a proteolytic cleavage in endothelial cells and vascular tissues in the presence of Asp298. This phenomenon can explain the vascular NO production deficit in T allele carriers (25).

Although in many studies it has been referred to the fact that the T allele of this gene polymorphism results in the production of deficient protein and the reduction of its enzymatic activity which can be a risky allele for ESRD (12, 17, 26-27), other studies did not prove the significant differences between Glu298 and 298Asp functions (28- 29). Thus there is still discrepancy over the fact that whether T allele plays a modifier role in eNOS activity and the decrease of the ESRD age in ADPKD patients. 

In a study by Jung Geon Lee et al. (2002), a total of 112 Korean ADPKD patients and 41 non-patients were genotyped by PCR-RFLP, The distribution of the alleles for the Glu298Asp polymorphism in ADPKD patients under study was 88% and 12% for G and T respectively. Their results suggest that the polymorphism at Glu298Asp of ecNOS had no association with the renal progression in Korean ADPKD patients (29). The frequency of NOS3 gene genotypes in the present study showed that this study is in line with the former study and doesn’t present any statistically significant difference for Glu298Asp polymorphism among the investigated groups. In order to explore the effect of this polymorphism on the lower age at ESRD in ADPKD patients, Walker et al. (2003), described the same polymorphism in 215 mutation-defined polycystic kidney disease 1 (PKD1) patients among 80 families. They concluded that the Asp 298 eNOS allele may be associated with lower vascular activity of eNOS, but this did not correlate with severity of renal disease in the population inflicted with PKD1(28). Similarly, the present study categorized individuals based on their needs to renal replacement therapy.

 In our study, age of ESRD is the age when the patient has undergone dialysis or transplant for the first time. Like Walker's study, the results for Glu298Asp Polymorphism genotype distribution showed that there was no significant relationship between the polymorphism and the decrease in age of ESRD onset. However, A. Persu et al. (2002), research on ADPKD patients in Belgium and the north of France was paradoxical. They described the age at ESRD as the age at starting renal replacement therapy. The ESRD age ranged from 50 to 52 in these patients. According to the data,the frequent Glu298Asp polymorphysim was associated with a 5 year lower mean age at ESRD in ADPKD males (18). Also the study of N. Stefanakis et al (2008) on 100 Greek ADPKD patients and 107 healthy individuals indicated that the T allele of the Glu298Asp polymorphism of NOS3 gene is associated with earlier progression to ESRD in ADPKD patients. The difference observed between our data and some other investigations could be due to the limitation of the size of the population we studied.

Since this polymorphism can affect the blood pressure and the Glomerular filtration rate by changing the vascular endothelial functions such as renal's arteries and thus cause the progression of the disease and also due to the importance of clinical symptoms in estimating the progression of ADPKD, the investigation of the relationship between this polymorphism and the clinical features related to the disease will gain significance. According to the present study Glu298Asp polymorphysim had no significant effect on serum creatinine level and blood pressure, among the wild type and mutated groups. Moreover clinical parameters related to ADPKD were also investigated. These parameters include: hematuria, kidney stones, BUN, Plt, WBC, HDL/LDL, FBS, hemoglobin, sodium, potassium, calcium, phosphorus, triglyceride, and cholesterol. However, since no significant difference in the distribution of T and G alleles was observed for any of the above mentioned factors in normal or abnormal conditions, this polymorphysim cannot be an intervening factor in the investigated population. It is worth mentioning that given the limited scope of the previous studies, the present study is significant in that it focuses on a wider range of data. Due to the limitation of the investigated factors in similar studies in comparison to the present study, the generalization of results for these variables is not possible.

## Conclusion

Glu298Asp polymorphysim of NOS3 gene was not related to decreasing ESRD age in ADPKD patients in this population. Also T allele in position 894 of this gene dose not seem to be a risk factor for progression of ADPKD.
